# Implementation Strategies for Interventions Aiming to Increase Participation in Mail-Out Bowel Cancer Screening Programs: A Realist Review

**DOI:** 10.3389/fonc.2020.543732

**Published:** 2020-09-29

**Authors:** Larry Myers, Belinda Goodwin, Nicholas Ralph, Oscar Castro, Sonja March

**Affiliations:** ^1^Centre for Health, Informatics, and Economic Research, University of Southern Queensland, Springfield Central, QLD, Australia; ^2^School of Psychology and Counselling, University of Southern Queensland, Springfield Central, QLD, Australia; ^3^Cancer Research Centre, Cancer Council Queensland, Brisbane, QLD, Australia; ^4^School of Nursing & Midwifery, University of Southern Queensland, Toowoomba, QLD, Australia; ^5^Faculty of Health, University of Technology Sydney, Ultimo, NSW, Australia; ^6^Physically Active Lifestyles Research Group, Institute for Resilient Regions, University of Southern Queensland, Springfield Central, QLD, Australia

**Keywords:** bowel cancer screening, HAPA, behavior change techniques (BCTs), realist review/synthesis, interventions

## Abstract

**Background:** Bowel cancer is the third most commonly diagnosed cancer and the third most common cause of cancer-related death, with 1,849,518 new cases of bowel diagnosed and 880,792 deaths reported globally in 2018 alone. Survival can be improved through early detection via national mail-out bowel cancer screening programs; however, participation remains low in many countries. Behavior change is therefore required to increase participation. This realist review aims to (a) identify the behavior change techniques (BCTs) used in each intervention, (b) understand the mechanisms of action (MoAs) responsible for the BCT effectiveness, and (c) apply a behavior change model to inform how MoAs can be combined to increase screening participation.

**Methods:** We systematically reviewed the literature for interventions aiming to increase participation in mail-out bowel cancer screening. We used a four-stage realist synthesis approach whereby (1) interventions were extracted from each study; (2) BCTs applied in each intervention were identified and coded using the BCT Taxonomy-v1; (3) the Theory and Techniques Tool was used to link BCTs to their MoA; and (4) BCTs and MoAs were categorized according to their effectiveness and what Health Action Process Approach (HAPA) stage of change they would affect.

**Results:** We identified 68 intervention trials using 26 unique BCTs and 13 MoAs to increase participation. Sixteen BCTs and 10 MoAs were identified within the interventions that successfully increased participation rates. Interventions targeting both stages of the HAPA model had a higher success rate (80%) than those targeting one stage of change (51%). When targeting only one stage, interventions targeting the volitional stage had a higher success rate (71%) than interventions targeting only the motivational stage of change (26%).

**Conclusion:** Importantly, this review identified a suite of BCTs and MoAs effective for increasing participation in mail-out bowel cancer screening programs. With increased participation in bowel cancer screening leading to improved survival, our findings are key to informing the improvement of policy and interventions that aim to increase screening using specific strategies at key stages of health decision-making.

## Introduction

Bowel cancer has the third-highest incidence rate and the third-highest mortality rate of all cancers worldwide (1). If detected early enough, ~90% of cases are cured (2). To aid in early detection, population-based screening is now commonplace in developed countries. At least 24 countries have now adopted national bowel cancer screening programs including Australia, Canada, and the United Kingdom, with fecal occult blood testing (FOBT) the most effective population screening tool for detecting early signs of bowel cancer (2–4).

Typically, FOBT kits are sent directly to the recipient's homes (4). Invitees are asked to collect small stool samples using the FOBT kit provided and mail the samples back for processing (5). It is recommended that those older than 50 years (i.e., the average-risk population) do this once every 2 years (4). If the test is positive, the individual is then referred to further diagnostic tests such as colonoscopy and biopsy (6, 7). This two-stage process is highly cost-effective and sensitive at detecting bowel cancer (8, 9).

Nevertheless, low participation in FOBT screening is frequently reported, with countries such as Australia, France, Czech Republic, Germany, Latvia, and Croatia reporting fewer than one in two invitees return the test (4, 6, 10, 11). Correspondingly, bowel cancer mortality remains disproportionately high in these countries, in part due to poor screening uptake and later diagnosis and treatment (12). Increasing participation is therefore a common focus in the literature with a range of interventions trialed. Findings from two recent systematic reviews (13, 14) highlight four key implications for improving implementation of bowel cancer screening programs:

(a) Some interventions consistently increase participation rates [e.g., advance notification letters, simplified testing procedures, telephone contact, and use of general practitioner (GP) endorsement], but their effects are small to moderate (13).
(b) There are large levels of heterogeneity in these effects due to the variation in implementation (13).
(c) Using multiple intervention strategies is associated with larger effects (14).
(d) Little is known about how these interventions work, and an overarching framework for how interventions should be combined has yet to be established (14).

These heterogeneous and modest intervention effects are unsurprising, given the large variation in the reasons provided by invitees for non-participation. Reasons for non-participation are diverse and include (but are not limited to) emotional disgust in the process, seeing the test as unnecessary, procrastination, and fear of a cancer diagnosis (15–17). Studies have also noted distinct groups of people within those choosing not to participate in FOBT screening, those who have no motivation to do the test all together, and those who intend to do the test but do not, often due to procrastination, forgetting, or inconvenience (15, 16). Thus, for interventions to be effective in population-based screening programs, they need to overcome various and multiple barriers to have the greatest effect and facilitate screening for distinct groups of people (14). This can be systematically accomplished by establishing a comprehensive behavior change framework to address the nuances of non-participation in FOBT screening programs.

Policymakers and organizers of mail-out FOBT screening programs must make use of suitable evidence-based interventions to increase participation. However, evidence gaps are hindering these efforts. First, differences in how interventions are described in the literature make it difficult to decide which elements are the “active ingredients” (otherwise known as behavior change techniques) and should be incorporated into national screening programs. For example, two separate interventions to increase FOBT kit use provided an endorsement letter from the invitee's personal GP (18, 19). Although seemingly similar, one letter focused on delivering health messages as the endorsement (18), whereas the other acted only as a reminder to return the kit (19), with only the former significantly increasing participation rates. This demonstrates the need to go beyond assessing if an intervention as a whole can significantly increase participation rates, to identifying and evaluating the individual intervention components that are responsible for behavior change. In this manner, the most effective intervention components can be established and implemented within national bowel cancer screening programs.

Second, knowing that an intervention component can bring about behavior change does not necessarily assist in identifying the underlying behavioral mechanisms responsible for the behavior change; with these being known as mechanisms of action (20). These mechanisms of action can be seen as the mediating factor between the intervention itself and the change in behavior (21). It is important to understand the mechanisms of action by which the interventions work so adaptations can be made to fit the given context and effectively design new interventions (21). Identifying the effective mechanisms of action is of additional importance in the context of FOBT screening as these interventions predominately involve sending extra information to the invitees. Previous research has shown that an overload of the information sent to invitees can result in a decrease in FOBT screening participation (22). It is therefore important to make the most efficient use of any materials sent to invitees. For example, providing health information about bowel cancer as an intervention may work through multiple mechanisms, such as increasing the invitee's perception of risk of developing the disease and/or increasing the invitee's belief that he/she can take preventive action. If only one of these mechanisms is likely to bring about behavior change, interventions should focus on delivering messages that evoke that one mechanism and disregard superfluous information-based interventions that may lead to an information burden that produces a counteractive effect.

One framework that has been constructed to address these issues of intervention reporting and discerning their related mechanism of action is the combined use of the behavior change techniques Taxonomy-v1 (23) and the Theory and Techniques Tool (21, 24). The behavior change techniques Taxonomy-v1 is a comprehensive list of behavior change techniques that have been trailed in health behavior interventions. It was designed to create an agreed-upon language that can be used to describe the active components within interventions (23). These behavior change techniques can be linked to certain mechanisms of action using the Theory and Techniques Tool (25). Mechanisms of action describe the process by which these behavior change techniques bring about behavior change (21, 24). This combined framework allows for a systematic and reliable way to describe the active elements within intervention strategies and understand *how* interventions bring about behavior change.

It is also important to consider strategies within a larger theoretical framework to identify which behavior change techniques and mechanisms of action can be used to construct an effective multifaceted intervention. When designing new and effective behavior change strategies, research has shown that interventions based on psychological theory can be more effective than interventions that have no theoretical bases, with those that target multiple constructs within these theories being even more effective (26). One prominent framework is the Health Action Process Approach (HAPA), which models behavior change as two stages: first, a motivational stage where people develop intentions to engage in a behavior and then a volitional stage where people translate these intentions into behavior (27). For example, in the context of FOBT use, recipients who refuse to participate would be described as being in the motivational stage, whereas those who wish to screen but have not because of procrastination would be described as being in the volitional stage. According to the HAPA model, behavior change techniques and mechanisms of action can work synergistically when they facilitate change across both the motivational and volitional stages of change (28). To date, trials that have combined intervention strategies (i.e., multiple behavior change techniques and mechanisms of action) did so without reporting any theoretical grounds for combining those specific interventions together (14). Greater use of behavior change theory could assist in developing an effective intervention strategy that could bring about substantial improvements in bowel cancer screening participation and subsequently reduce the burden associated with this disease.

## Aims

This realist review aims to understand the behavioral mechanisms that are effective in increasing screening participation and identify what combination of behavior change techniques might work most effectively. Specifically, this review will identify all trials that reported on an intervention aiming to increase participation in mail-out FOBT screening programs. The objectives are as follows:

1. To identify the specific behavior change techniques that have successfully been used within interventions
2. To link these behavior change techniques with mechanisms of action to understand the potential process of behavior change in screening participation
3. To use the HAPA stages of change to examine what combinations of behavior change techniques and mechanisms of action tend to be effective.

## Methods

The current research aims were addressed using a realist synthesis methodology. Rather than focusing on making judgments about if an intervention works (such as traditional systematic reviews and/or meta-analysis), a realist review is more explanatory in nature and uses a generative model to infer how an intervention brings about behavior change [for a full description of this technique, see Pawson et al. (29)]. Realist reviews go beyond the question of what works to “what is it about this program that works for whom, in what circumstances?” (p. 22, 29). Consequently, findings from realist reviews tend not to be concise, such as meta-analytic point estimates, but rather the findings are complex and intricate and holistically address the multifactorial nature of health behaviors (30).

**Figure 1:**
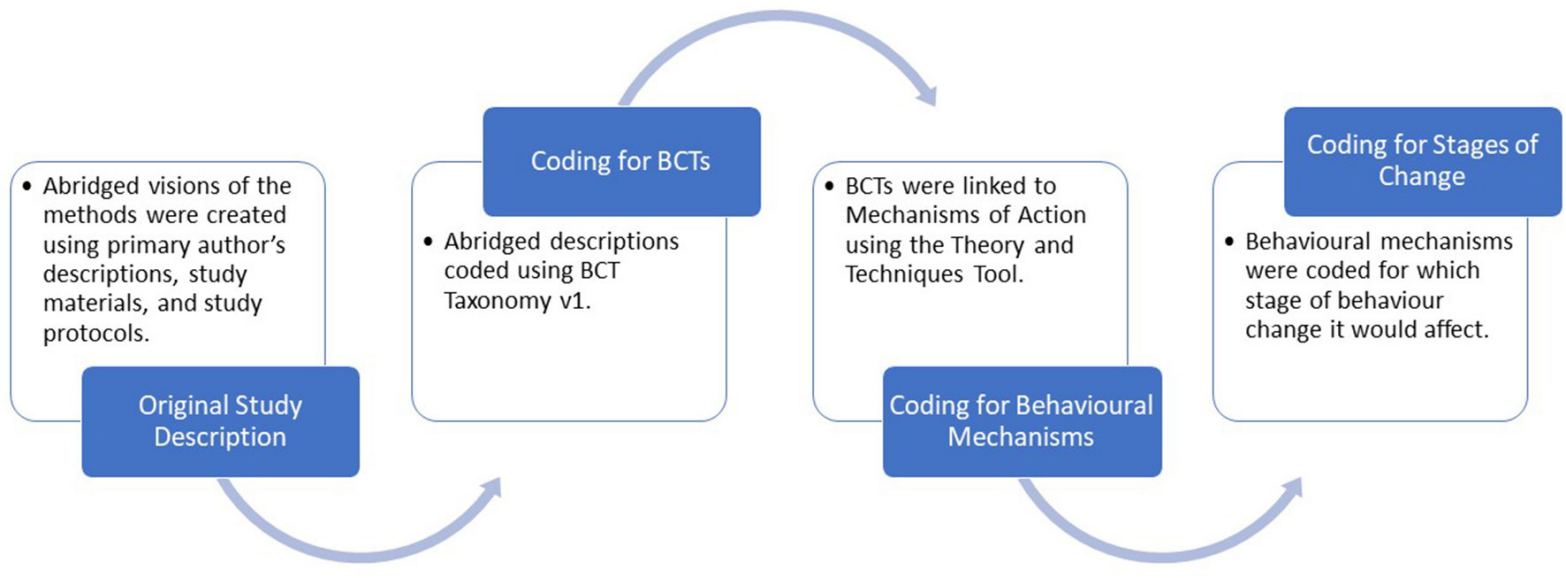
Four-stage realist synthesis.

The Realist and Meta-narrative Evidence Synthesis: Evolving Standards (RAMESES) guidelines were used to conduct this review (31). See Supplementary Material 1 for the RAMESES II reporting standards for the realist evaluations checklist. After the systematic search process was complete, we adapted a novel four-stage realist synthesis approach to identify what makes an intervention successful at increasing participation in mail-out FOBT screening programs and how they bring about behavior change (depicted in Figure 1).

### Search Strategy and Screening

The search strategy followed the same procedure as a 2018 systematic review of interventions aiming to increase participation in mail-out FOBT screening (13) with an updated date range to include dates up to June 20, 2019. Included studies (a) reported on interventions aimed at improving participation in mail-out bowel cancer screening, (b) involved the mailing of a screening kit to the participants' homes without a specific request from the individual, and (c) included quantitative data that reported on the FOBT return rate.

Studies were excluded if (a) the screening kit was not mailed directly to the participant, (b) studies required participants to request a kit or accept an invitation to receive a kit in the future or to be part of the study, (c) studies investigated other types of bowel cancer screening (e.g., colonoscopy) and did not report specific outcomes for FOBT screening, and (d) the full text was not available in English.

**Figure 2:**
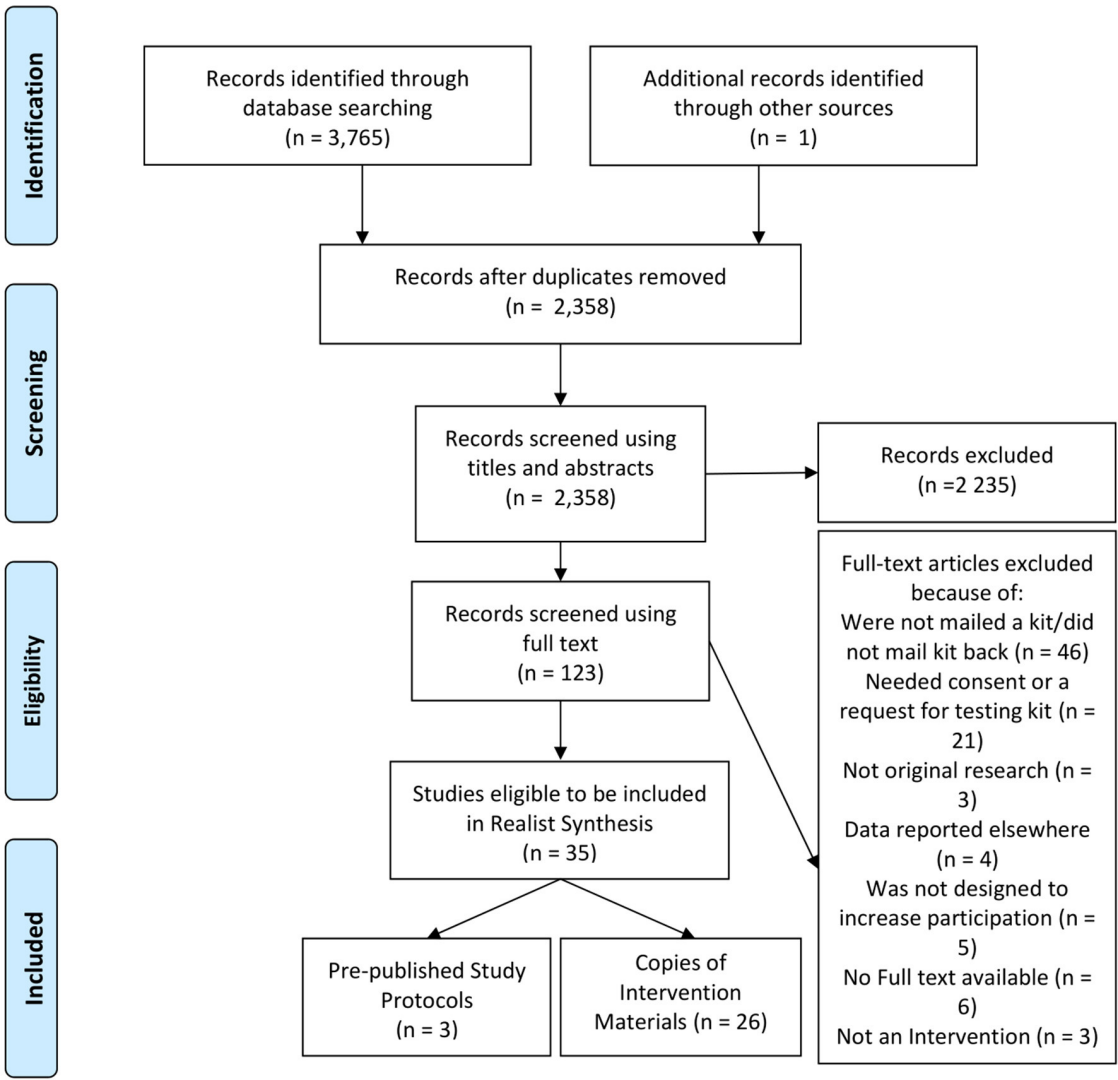
Document flow chart of systematic search.

These searches were conducted with six databases; PubMed, Scopus, PsycINFO, CINAHL, Google Scholar, and Proquest Theses and Dissertations. See Figure 2 for document flowchart and Supplementary File 2 for detailed search strategy (13, 14).

### Stage 1: Data Extraction

For each study, short descriptions of the procedures and intervention materials were extracted. When available, these descriptions were further informed by study protocols and online versions of the materials. In cases where this information was not readily available, best efforts were made to contact the original authors for a copy of their materials.

### Stage 2: Coding Behavior Change Techniques

To address the first research objective, stage 2 identified what techniques were used within interventions to affect participation rates, using the behavior change techniques Taxonomy-v1 (19). The behavior change technique Taxonomy-v1 contains 93 non-redundant behavior change techniques (e.g., the behavior change techniques “imaginary punishment,” “imaginary reward,” and “vicarious consequences”). Each behavior change technique has a unique definition that describes an “observable, replicable, and irreducible component of an intervention designed to alter or redirect causal processes that regulate behavior” (p. 23, 23). By using these definitions, and the instructions available at the website (https://www.bct-taxonomy.com), each behavior change techniques present within each intervention were identified. Behavior change technique identifications were made from the description within the methods section of each article and when possible the intervention materials themselves. Behavior change techniques that were part of “usual care,” as opposed to being part of an intervention strategy, were not recorded or analyzed.

### Stage 3: Linking Behavior Change Techniques to Mechanisms of Action

The purpose of stage 3 was to address the second research objective: to understand the process by which these behavior change techniques did and did not bring about behavior change (21). The Theory and Techniques Tool was used to link behavior change techniques identified in stage 2 to their mechanism of action (21, 24). The Theory and Techniques Tool suggests links between 74 behavior change techniques and 26 mechanisms of action. These links were established through a synthesis of research literature and consensus of experts in the field of behavior change (21, 24). In the Theory and Techniques Tool, each mechanism of action may have one or more linked behavior change techniques with varying evidence for the suggested link (e.g., the mechanism of action “reinforcement” has a link to the behavior change technique “material incentive,” an inconclusive link to the behavior change technique “associative learning,” and a non-link to the behavior change technique “information about health consequences”). In the current review, the information and procedure provided by the Theories and Techniques tool along with the context of the given application were used to decide on a link between the behavior change techniques identified in stage 2 and their mechanisms of action.

### Stage 4: Identifying Stages of Behavior Change

Stage 4 addresses the third research objective and examines what combinations of behavior change techniques and mechanisms of action tend to be effective. Stage 4 used the HAPA model to categorize how the individual behavior change techniques and mechanisms of action might work synergistically to address the variety of barriers that occur at various stages of change experienced during the process of receiving, using, and returning an FOBT kit (32). The HAPA model posits a two-stage change relevant to CRC screening: (1) a motivational stage and (2) a volitional stage, with different factors being influential at the different stages. The model also includes factors related to maintenance and recovery of the health behavior; however, these are not relevant in the context of FOBT screening, a “one-off/occasional” behavior. The HAPA model suggests that people need to develop risk perceptions, outcome expectations, and task self-efficacy (i.e., the confidence person has in performing the action) to develop the motivation to engage in any health behavior. While factors such as action planning, coping planning, and maintenance self-efficacy (i.e., confidence the person has in overcoming barriers) are influential in the volitional stage. The HAPA model argues that interventions should first increase people's motivation (e.g., providing information regarding the benefits of bowel cancer screening) and then help the person translate this motivation into action (e.g., by developing useful action plans) (27). By using the HAPA model in conjunction with the Theory and Techniques Tool, it can be determined what mechanisms of action are effective for the different stages of change and which mechanisms of action should be combined so that both stages of change are targeted by an intervention strategy. Thus, in stage 4, the mechanisms of action within each intervention were coded according to whether they were likely to affect the motivational or volitional stage of change as described by the HAPA model.

### Coding, Synthesis, and Analysis of Findings

Researchers performed coding independently at all stages of the review (L.M. and O.C. for stage 2, L.M. and B.G. for stage 3, and L.M. for stage 4). All reviewers responsible for coding the intervention content for behavior change techniques have completed the online behavior change technique taxonomy training (“behavior change technique Taxonomy-v1 Online Training,” 2019). Discrepancies between reviewers at any of these stages were resolved by a consensus discussion with the wider research team.

Two methods were applied to synthesize findings. First, the behavior change techniques and mechanisms of action identified were grouped according to whether or not they were applied in an intervention trial that significantly increased participation rates. Comparisons were then made regarding the frequencies of behavior change techniques and mechanisms of action and how often they were part of a successful intervention. Second, individual trials were also grouped according to the HAPA model stage(s) the mechanisms of action in that intervention addressed and whether the intervention significantly increased participation rates. This was analyzed to descriptively examine if the HAPA model stage addressed by an intervention had any association with the likelihood of the intervention being successful at increasing participation rates.

## Results

### Stage 1 and Document Characteristics

As seen in Figure 2, 35 articles were found that met the inclusion and exclusion criteria. This included a total of 68 intervention trials. All 68 individual intervention trials were included in this analysis (see Supplementary File 3 for a summary of these interventions). In addition to the published articles, 11 studies (30.6%) had the intervention materials readily available, four studies (11.1%) gave these materials on request, seven studies (19.4%) could not provide the materials (due to lost files or language other than English), and 14 studies (38.9%) could not be contacted or did not respond to the request. In total, intervention materials were available for 26 trials. Three published protocols were also found relating to these studies (33–35).

Studies took place in eight different countries: United Kingdom (*n* = 10), Australia (*n* = 7), the Netherlands (*n* = 5), United States (*n* = 5), Scotland (*n* = 4), Israel (*n* = 2), Latvia (*n* = 1), and New Zealand (*n* = 1). A risk-of-bias assessment was conducted on this set of studies using the Cochrane Risk of Bias tools (36–38), and methods are detailed in previous systematic reviews (13, 14). Briefly, 17 articles were of low risk of bias (22, 33, 34, 39–51), three studies were of moderate risk of bias (52–54), nine studies were of unclear risk of bias (55–63), four were of high risk of bias (64–67), and two articles were of serious risk of bias (68, 69).

### Stage 2: Behavior Change Techniques

**Figure 3:**
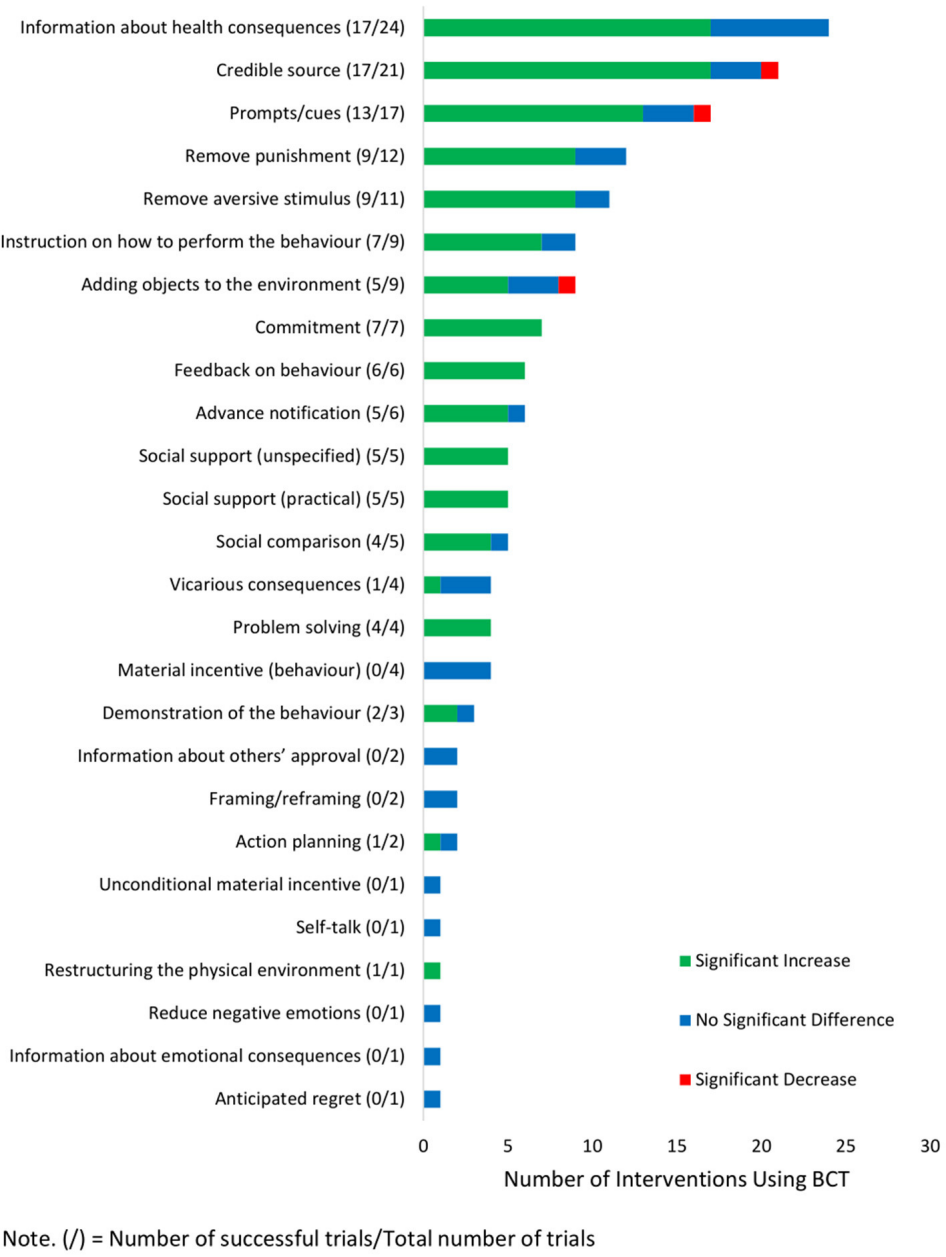
Behavior change techniques identified across interventions.

Across the 68 interventions, 26 unique behavior change techniques were identified with the frequency of use displayed in Figure 3. Overall, this review found that there was no single behavior change technique that could be recommended as a necessary component to be implemented in all mail-out FOBT programs. Rather, this review found a suite of behavior change techniques that are flexible in their implementation, can be part of an effective strategy, and should be utilized together. For instance, the most frequently used behavior change technique was the provision of “information about health consequences” (*n* = 24). Depending on the information provided, this behavior change technique changed behavior through two distinct mechanisms: informing people about the risks of bowel cancer (mechanism of action “perceived susceptibility”) and/or informing people of the reduced risks if they participate in the program (mechanism of action “beliefs about consequences”). This behavior change technique was often used in conjunction with the second and third most frequently identified behavior change techniques: delivering messages from a “credible source” (e.g., personal GP or health network; mechanism of action “attitude toward the behavior,” *n* = 21) and issuing “prompts/cues” (*n* = 17) to remind invitees to complete and return the kit (mechanism of action “behavioral cueing”). While a large proportion of interventions that used these three behavior change techniques were successful (Figure 3), ~20–30% of these trials did not increase participation rates. This suggests that the frequently used behavior change techniques “information about health consequences,” “credible source,” and “prompts/cues” often are, and should be, part of an effective strategy; however, there are circumstances under which they may not bring about increases in FOBT participation.

As seen in Figure 3, six behavior change techniques were associated with increased participation rates in 100% of their uses and are strong candidates to be implemented in FOBT screening programs. All interventions that included the following significantly increased participation: had invitees make a “commitment” (*n* = 7) to return the completed FOBT, gave “feedback on behavior” (*n* = 6) that the invitee had yet to return the FOBT kit, used live “social support (practical)” (*n* = 5) to give instructions on how to complete the FOBT kit, used live “social support (unspecified)” (*n* = 5) to encourage people to complete the FOBT kit, had invitees engage in “problem-solving” (*n* = 4) to overcome barriers associated with FOBT participation, and involved “restructuring the physical environment” (*n* = 1) by accepting completed FOBT kits in community drop-off locations as well as mailed returns.

However, several caveats need to be considered before implementing the aforementioned behavior change techniques. “Restructuring the physical environment” has only been trialed once, and replication of the finding is needed before its efficacy can be established (52). The remaining five of these behavior change techniques, “commitment,” “feedback on behavior,” “social support (practical),” “social support (unspecified),” and “problem-solving,” were predominately delivered using live telephone calls. While the relatively small samples in these studies (*n* < 590) meant that this was feasible (41, 49, 59), national screening programs typically send millions of FOBT kits every year (6). Thus, employing a strategy that requires live telephone calls may not be scalable. Nonetheless, these behavior change techniques do show promise in controlled contexts, and future research should focus on how to implement these key components on a larger scale.

An important finding from this review was that 69 behavior change techniques listed in the behavior change technique Taxonomy-v1 were not trialed in any of the reviewed studies. This provides a significant opportunity to create novel intervention strategies. For example, behavior change techniques such as having invitees create a “pros and cons” list, sign a “behavioral contract,” or increasing the “salience of consequences” have yet to be trialed in the context of mail-out FOBT screening and are possible avenues for future research to increase participation. When deciding which novel behavior change techniques to trial researchers should base their judgments on the known barriers to screening (15, 16, 70) and what behavior change techniques can be applied in the context of mail-out FOBT screening.

It should be noted that two behavior change techniques did not fit any of the descriptions within the behavior change technique Taxonomy-v1 and were designated their own categories. One of these strategies involved notifying invitees weeks prior that an FOBT kit will be arriving soon (44, 49, 55, 61, 62). This was labeled “advance notification” (44, 49, 55, 61, 62). The other strategy involved sending a $10 gift voucher with the FOBT invitation (not conditional on FOBT completion) as an incentive to complete the FOBT kit (51). This was labeled “unconditional material reinforcement.”

### Stage 3: Linked Mechanisms of Action

**Figure 4:**
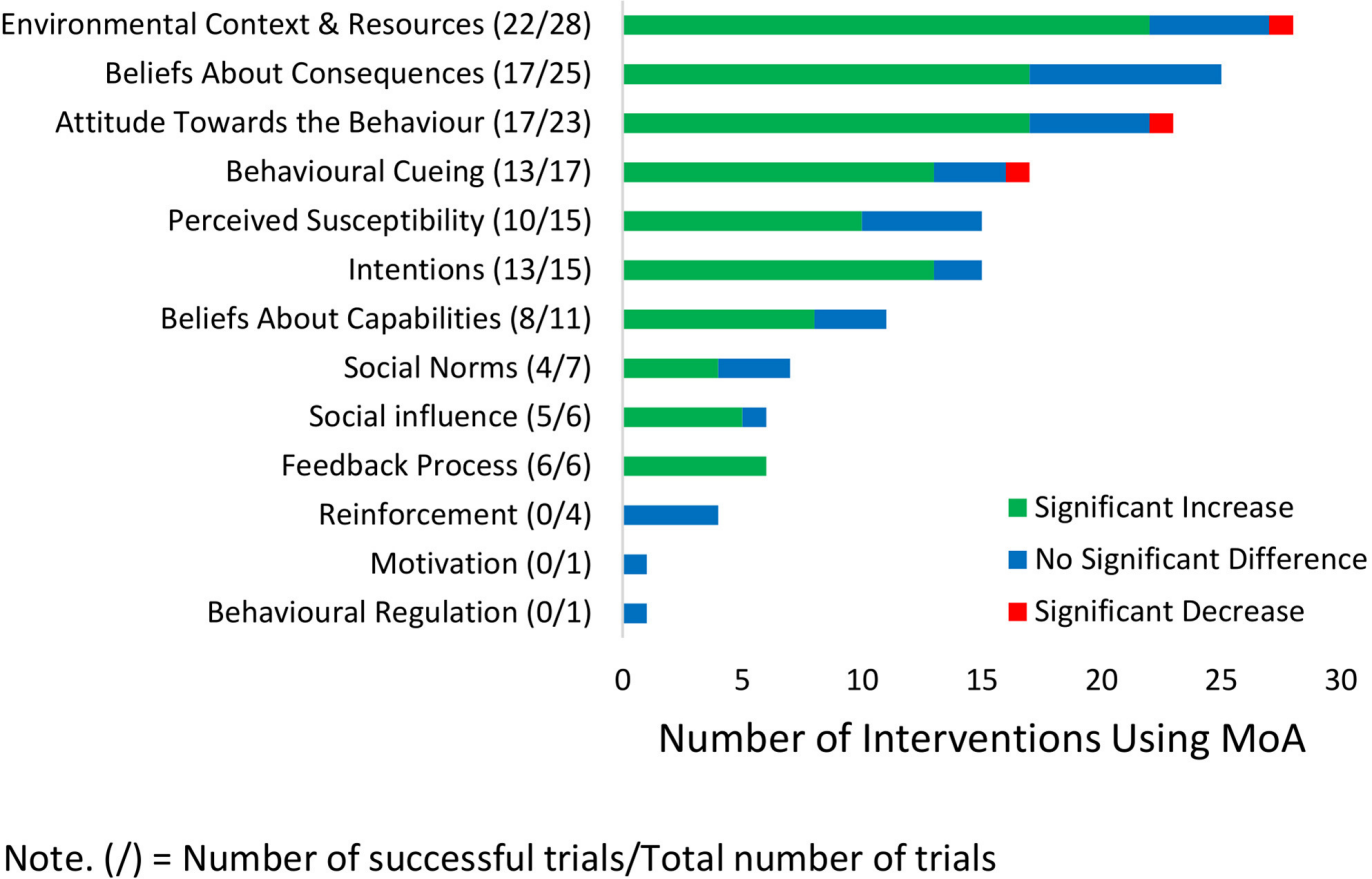
Mechanisms of action identified across interventions.

In total, 13 different mechanisms of action were linked to the behavior change techniques utilized in the given interventions; these are displayed in Figure 4. The most commonly employed mechanism of action was to change the “environmental context and resources” (*n* = 28) available to the invitee, which reduced the barriers commonly related to FOBT screening. This mechanism of action was successful in 78.6% of cases, with differences in implementation appearing to drive the variability in efficacy. The behavior change techniques that successfully used this mechanism of action to increase participation rates did so by reducing the number of screening tests needed (behavior change technique; “remove aversive stimuli”) (e.g., 43, 70) and/or removing the need for any dietary restriction (behavior change technique; “remove punishment”) (e.g., 41, 66, 72). Overall, the efficacy of the mechanism of action changing the “environmental context and resources” predominately reflects the success of programs that have switched to a newer FOBT kit [known as a fecal immunochemical test (FIT)] that require fewer samples and no dietary restrictions. However, many countries already use these newer FIT kits, and participation rates are still in need of improvement. Additional strategies will therefore be needed to increase participation rates.

In contrast, studies that changed the “environmental context and resources” by making the sampling procedure easier (e.g., by use of collection aids; behavior change technique “adding object to the environment”) either did not increase participation rates or was only successful when delivered with other mechanism of action (56, 67). As the sampling procedure itself is a reported barrier to screening (15, 16), further investigation is needed to improve the design of the screening kits to facilitate participation.

As screening programs may have limited capacity to change the FOBT kit (or the aforementioned effective changes have already been made), other mechanisms of action can be used to increase participation rates and do not require changing the testing kit or procedure. One of these mechanisms of action that are highly effective is to use “behavioral cuing” to remind/prompt invitees to complete the FOBT. This can be successfully implemented through various mediums such as media campaigns, live telephone calls, or direct mailed reminders (41, 54, 64). Media campaigns, in particular, have been shown to be a cost-effective way to deliver these messages (54, 67, 71); however, there is some evidence to suggest that these campaigns need to be of high intensity (i.e., multiple mediums over multiple times) to be effective (54). It should be cautioned that those interventions that used text messages or automated phone calls as a medium for their “behavioral cuing” did not increase participation (19, 41). These findings suggest that prompts and cues should be delivered using media campaigns, live telephone calls, or direct mailed reminders to be effective.

Helping people develop “intentions” was also a mechanism of action that was frequently associated with increased participation rates. This was done either implicitly, through sending letters weeks in advance of the testing kit instructing the invitee in what to do when it arrives (behavior change technique “advance notification”), or explicitly, through asking invitees during a phone call to verbally commit to completing the kit (behavior change technique “commitment”) or having invitees set a time and date for when they will do the test (behavior change technique “action planning”). This latter behavior change technique of “action planning” was successful only when the invitees completed their own action plan; when the same strategy was used but with prefilled responses to the planning questions, no difference was found in participation (46, 58). Screening programs that enhance intentions and help invitees create their own specific plans and commitments seem more likely to be effective.

Providing feedback to the invitee that they have not completed their test (mechanism of action “feedback process”; behavior change technique “feedback on behavior,” *n* = 6) is recommended to be included in screening programs as it was a highly successful mechanism and was the only mechanism of action that was associated with increased participation in 100% of its trials. Again, this was predominantly done through live telephone calls, which limit the potential scalability. However, this mechanism of action has also been coupled with a reminder letter (mechanism of action “behavioral cuing”) and that implementation successfully increased participation rates (64). This may provide a way to implement this highly successful mechanism at the scale of a national screening program.

A further 13 behavioral mechanisms are listed in the Theory and Techniques Tool and were not trialed in any of these interventions. However, 12 of these mechanisms of action either appear not to be applicable (e.g., “knowledge and existence of something” and “skill acquired through practice”) or similar mechanisms of action have been trialed instead (e.g., the mechanism of action “norms” has not been trialed, but “social norms” has been trialed). This suggests that most mechanisms of action have been trialed, and research should focus on new behavior change techniques and combinations of behavior change techniques to better engage the mechanisms of action that have been found to be effective. One untried mechanism of action that may be effective is the use of invitee's “self-image” (one's conception and evaluation of oneself) to increase participation rates. Invitees often find the arrival of the FOBT kit as a negative reminder of their age, and this acts as a barrier to participation (15). Thus, informing invitees that FOBT screening is for the young and old may be a potential way to utilize the mechanism of action of “self-image” and reduce this barrier and increase participation.

### Stage 4: HAPA Stages

**Figure 5:**
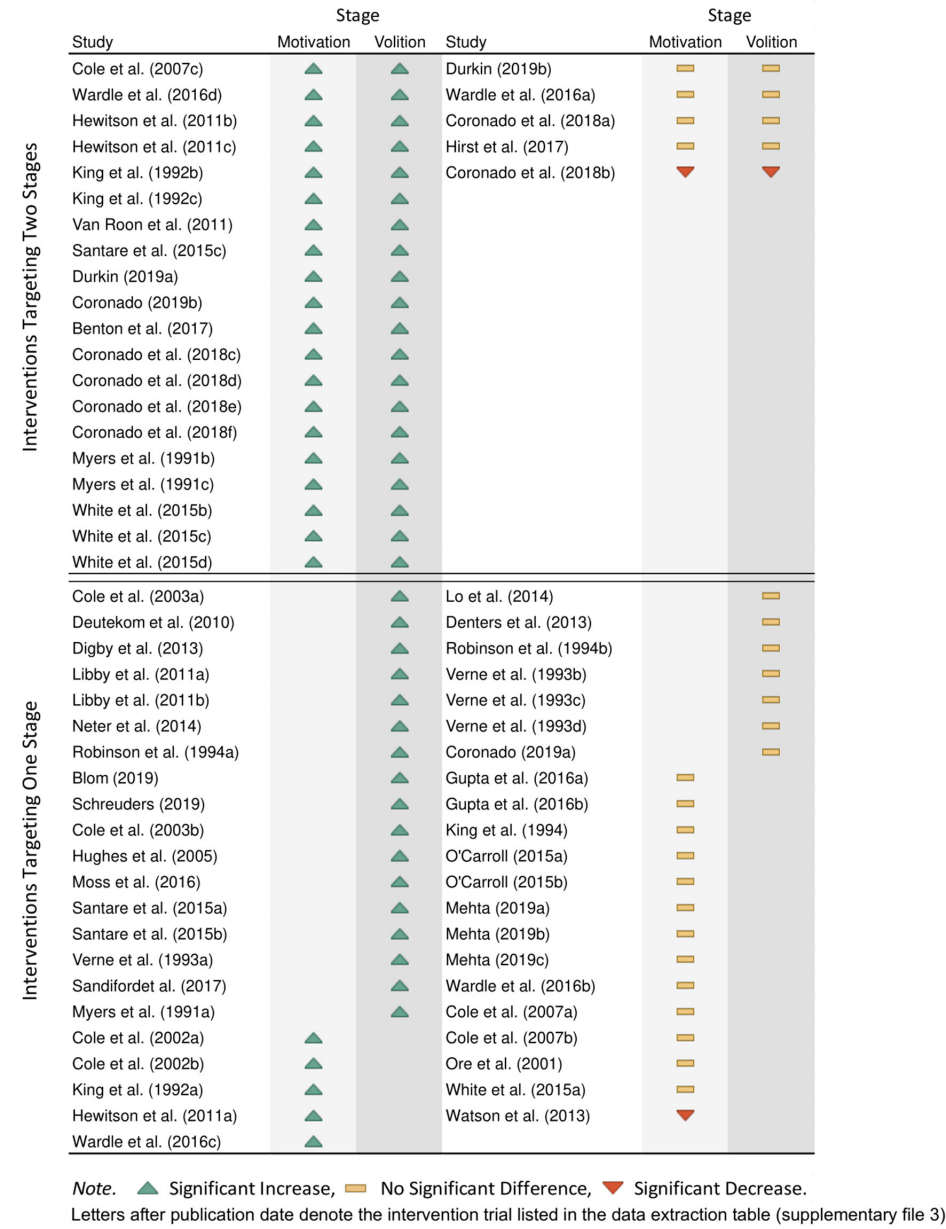
Stage of change targeted by each intervention.

Figure 5 shows a heat map of each intervention trial, which indicates the stage of change the trial targeted and if the trial significantly increased participation rates. As seen in Figure 5, 36.8% (*n* = 25) of interventions targeted both the motivation and volition stages together, 80% (*n* = 20) of which significantly increased participation rates. In contrast, 63.2% of trials targeted only one stage of change (*n* = 43), of which 51.2% (*n* = 22) significantly increased participation rates. These findings suggest that interventions should attempt to increase invitee's motivation to participate in screening as well as facilitate the screening process itself to maximize the likelihood of success. These findings are consistent with previous research that suggests interventions should be combined to enhance impact (14) and provides a framework for deciding which interventions should be combined and how.

Furthermore, the findings of this review are in line with previous qualitative findings that propose two distinct categories of non-participants in mail-out FOBT screening programs: those who intend to participate in the program but whose intentions have not yet translated into action (i.e., those in the volitional stage) and those who decide not to participate from the outset (i.e., those in the motivational stage) (15, 16). Interventions that target both the motivational stage and the volitional stage may be more likely to be effective because they facilitate change for both groups of invitees by addressing the specific barriers that are present for the distinct groups (27, 72).

When examining interventions that targeted only one stage of change, those that solely targeted the volitional stage had a higher success rate (70.8%, *n* = 17) than interventions that solely targeted the motivation stage (26.3%, *n* = 5). Past research has shown that strategies targeting the motivational stage may indeed be successful at increasing motivation to screen, but doing so will only move the participant along to a volitional stage where new barriers arise (such as the need for planning) (27). As such, interventions that only increase motivations do not necessarily help invitees overcome the new volitional barriers that arise when transforming motivations into action. Alternatively, intervention strategies that only target the volitional stage are likely to be successful in progressing those already with strong motivations to screen (i.e., those in the volitional stage) through to test completion, thus deeming the intervention successful (73). It is important to note that while volitional interventions appear more efficacious, there are still many people in the target population who do not have the motivation to screen because they misunderstand the risks of bowel cancer and/or the need for medical screening for early detection (15, 16). As such, both motivational and volitional interventions are needed to overcome barriers for the entire change process.

## Discussion

By identifying effective strategies for increasing participation, findings from this review address a key gap in the literature and provide a platform for implementing interventions that increase chronic low participation rates in bowel cancer screening programs across the world. We found strategies that increased participation predominately do so by (1) changing the resources available to reduce the burden of participation, (2) changing invitees' beliefs about the consequences of screening and their perceived risk of developing bowel cancer; and (3) providing effective cues. The specific behavior change techniques that were most consistently associated with increases in participation in mail-out FOBT screening programs included providing information about the health risk of bowel cancer, using credible sources to deliver these health messages, providing prompts or cues to remind people to complete the test, and changing to a FIT kit to reduce the number of samples needed and removing dietary restrictions. Importantly, interventions that increase motivations to screen as well as facilitate the screening process itself are most likely to be successful.

**Figure 6:**
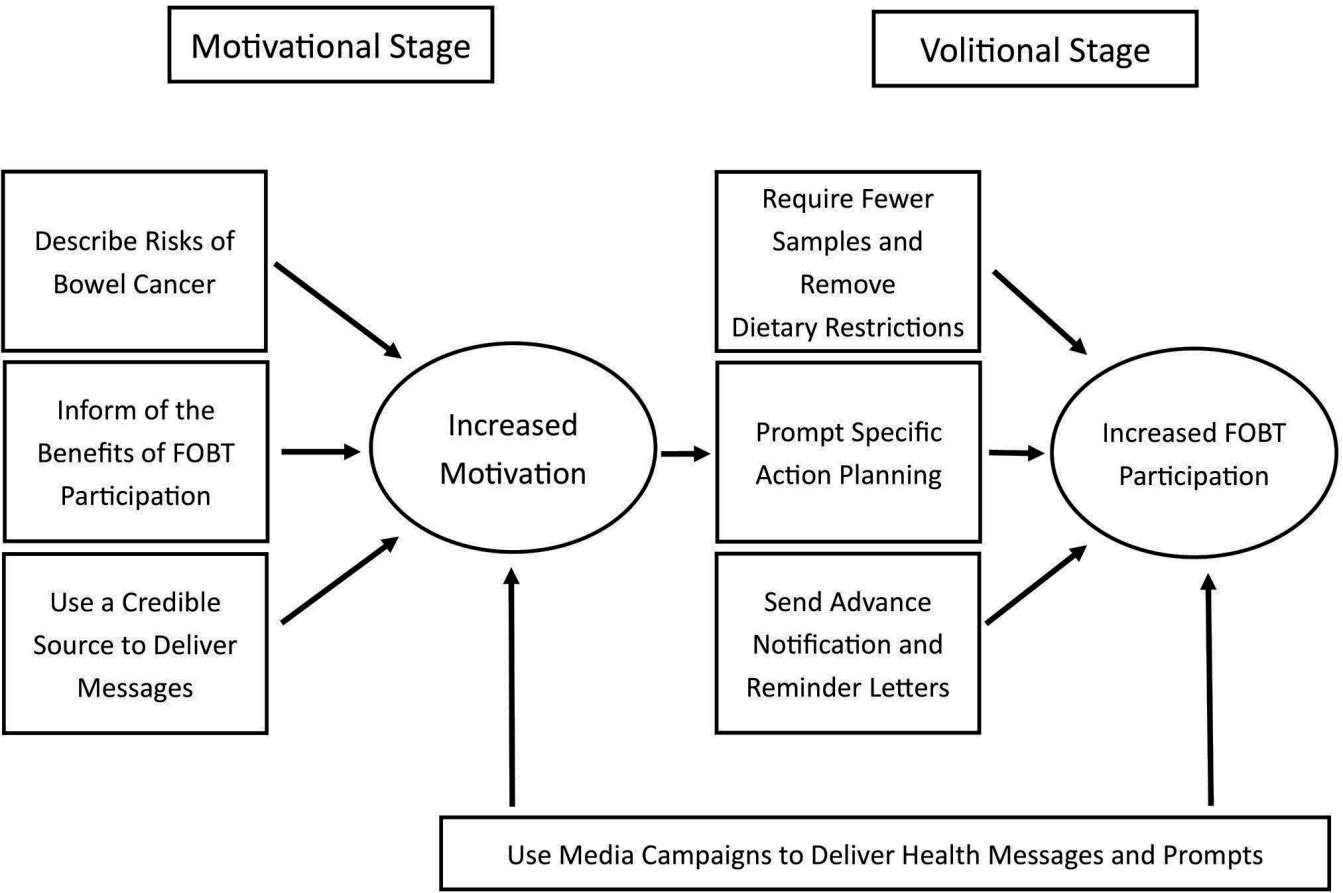
Proposed behavior change framework from current findings.

It is clear that there is no “one-size-fits-all” solution to increase participation rates in mail-out FOBT screening programs. Additionally, the exact type of intervention strategy adopted by screening programs will depend on what currently exists within the screening program itself. However, the findings from this review can be used to guide policymakers in their decisions as to which behavior change strategies should be implemented and combined to increase participation rates (Figure 6).

First, FOBT screening programs should aim to implement strategies that sufficiently motivate people to participate. To do this, the findings from this study suggest that messages should inform invitees of the high risks associated with bowel cancer, as well as how FOBT screening reduces these risks. According to the HAPA model, it is crucial that both these risk perception messages and positive outcome messages are included to sufficiently create motivation (27). Further, findings from this study also suggest additional strategies may successfully enhance the motivational power of such health messages. Motivations can be boosted if the health-related messages come from a trusted health professional, ideally from the invitee's personal GP (18, 64, 74). While this information can be sent directly to the invitee along with the FOBT kit, these motivational strategies can also be supplemented by large-scale media campaigns (54, 67).

Second, and in line with the assertions of the HAPA model, the findings of this study show that only increasing motivation to screen will not be optimal when attempting to impact participation rates. Rather, FOBT screening programs should also implement strategies that facilitate the transition of these motivations into action (i.e., completing and returning the FOBT kit). Specifically, intervention strategies should aim to help invitees overcome barriers (often through the reduction of barriers) and enable the creation of specific action plans for preforming the behavior (27). To address the barriers associated with FOBT screening, programs should supply the newer FIT kits that do not require any dietary restrictions and need fewer samples to be taken, while still being more accurate than FOBT (45, 75, 76). However, more research is needed on how to reduce barriers to fecal sampling in individuals eligible for screening. This has been noted as a barrier to participating, and previous attempts to improve the sampling procedure have not been effective (15, 56), thus representing a direction for future research. Additionally, helping people develop and commit to a specific action plan can reduce the number of people not participating due to procrastination or forgetting (77). The findings from this study show this can be done by prompting people to commit to a specific time and date for when they want to complete the test and having them set a plan for where they are going to keep the kit when it arrives (46). Finally, sending reminder letters to those who have not returned their FOBT kit can act as a type of feedback process and prompt more participation [e.g., (64)].

It should be cautioned that many of these behavior change strategies involve sending information to the invitee, and overloading invitees with information can reduce participation rates (22). As such informational messages should be spread across an advance notification letter, the invitation that includes the FOBT kit, and a reminder letter weeks after the FOBT kit's arrival. Not only have advance notification letters and reminder letters been shown to increase participation rates themselves (44, 55, 64), but also they give an opportunity to disperse the information load across time points reducing the risk of information burden hindering participation (22).

Accordingly, it is vital that comprehensive behavior change strategies are implemented to increase participation and that mail-out FOBT screening programs deliver a strategy that includes both motivational and volitional behavior change components. Policymakers can draw from the specific behavior change techniques and mechanisms of action highlighted in this review to guide new interventions to facilitate participation within their programs.

### Strengths and Limitations

This review is the first to examine what aspects of interventions are associated with increases in screening participation for mail-out FOBT screening. By making use of realist methodologies and a theory of behavior that models the distinct changes involved with FOBT screening participation, this review identifies the mechanisms that bring about behavior change and how these mechanisms relate to these distinct stages. Also, by using the behavior change technique Taxonomy-v1 and the Theory and Techniques Tool, the active elements and mechanisms of action of each intervention are able to be coded in a rigorous, transparent, and replicable manner aided by direct reference to intervention materials. Additionally, we ensured accuracy in coding the behavior change techniques from both published studies and intervention materials, through dual coding and coder training (78).

Nonetheless, study findings need to be interpreted with some limitations in mind, one being that many of the behavior change techniques were trialed within the same intervention, making it difficult to draw firm conclusions regarding the efficacy of each individual behavior change technique. While it can be identified that certain behavior change techniques form a part of a successful strategy when combined, it is not possible to infer with confidence which behavior change technique led to the intervention's success. Additionally, intervention materials were not available for all trials, so behavior change technique coding relied on the reporting in the original research article. As such, for these articles, it is possible some behavior change techniques were either not identified or misidentified.

## Conclusion

The results from this review present a range of behavior change techniques and mechanisms of action that, when included in an intervention, are likely to lead to increased participation rates in mail-out FOBT screening programs. Importantly, findings suggest that behavior change techniques and mechanisms of action should aim to increase invitee's motivation to participate in the screening program, as well as facilitate the translation of these motivations into active participation. Organizers of population mail-out FOBT screening programs should aim to identify which of the suggested behavior change techniques and mechanism of action are not already present within their programs and work to incorporate them such that all stages of change are targeted if they wish to improve participation rates.

## Data Availability Statement

Publicly available datasets were analyzed in this study. This data can be found here: In the Supplementary Material provided.

## Author Contributions

LM, BG, SM, OC, and NR made substantial contributions to developing the study and writing the manuscript. LM and OC coded the behavior change techniques. LM and BG coded the mechanisms of action. LM coded the HAPA framework. All authors contributed to the article and approved the submitted version.

## Conflict of Interest

The authors declare that the research was conducted in the absence of any commercial or financial relationships that could be construed as a potential conflict of interest.
