# Supplementary material for: Implementation Strategies for Interventions Aiming to Increase Participation in Mail-Out Bowel Cancer Screening Programs: A Realist Review
Source: Front Oncol. 2020 Sep 29;10:543732. doi: 10.3389/fonc.2020.543732 (PMC7550731; doi:10.3389/fonc.2020.543732)
Supplement: Supplementary file 1 [file Table_1.DOCX]

| Study | Trial Arm and Effect | Materials  Available | BCT | Behavioural Mechanism | HAPA Stage |
| --- | --- | --- | --- | --- | --- |
| Benton et al. (2017) | Reminder letter endorsed by personal GP. Significant increase in participation. | Yes | Prompts/cues | Behavioural cueing | Volition |
|  |  |  | Credible source | Attitude towards the behaviour | Motivation |
|  |  |  | Information about health consequences | Perceived susceptibility | Motivation |
|  |  |  | Social comparison | Social norms | Motivation |
|  |  |  | Feedback on behaviour | Feedback process | Volition |
|  |  |  | Information about health consequences | Beliefs about consequences | Motivation |
| Blom et al. (2019) | Using FIT with not dietary restrictions. Significant increase in participation. | No | Remove punishment | Environmental context & resources | Volition |
| Cole et al. (2002a) | GP endorsement letter by personal GP. Significant increase in participation. | No | Credible source | Attitude towards the behaviour | Motivation |
| Cole et al. (2002b) | GP endorsement letter by GP practice. Significant increase in participation. | No | Credible source | Attitude towards the behaviour | Motivation |
| Cole et al. (2003a) | Use of FlexSure FIT – removal of dietary restrictions. Significant increase in participation. | No | Remove punishment | Environmental context & resources | Volition |
| Cole et al. (2003b) | Use of InSure FIT – removal of dietary restrictions and fewer samples needed. Significant increase in participation. | No | Remove punishment | Environmental context & resources | Volition |
|  |  |  | Remove aversive stimulus | Environmental context & resources | Volition |
| Cole et al. (2007a) | Additional message about risk of CRC. No significant difference in participation | Yes | Information about health consequences | Perceived susceptibility | Motivation |
|  |  |  | Information about health consequences | Beliefs about consequences | Motivation |
| Cole et al. (2007b) | Advocacy for screening from previous screening programme participants. No significant difference in participation. | Yes | Information about others’ approval | Social norms | Motivation |
|  |  |  | Vicarious consequences | Beliefs about consequences | Motivation |
| Cole et al. (2007c) | Advance notification letter. Significant increase in participation. | Yes | Credible source | Attitude towards the behaviour | Motivation |
|  |  |  | Advance notification | Intentions | Volition |
| Coronado et al. (2018a) | Two automated phone calls reminders. No significant difference in participation. | Yes | Credible source | Attitude towards the behaviour | Motivation |
|  |  |  | Prompts/cues | Behavioural cueing | Volition |
| Coronado et al. (2018b) | Two text messages reminders. Significant decrease in participation. | Yes | Credible source | Attitude towards the behaviour | Motivation |
|  |  |  | Prompts/cues | Behavioural cueing | Volition |
| Coronado et al. (2018c) | A live phone call reminder. Significant increase in participation. | Yes | Social support (unspecified) | Social influence | Motivation |
|  |  |  | Social support (practical) | Environmental context &resources | Volition |
|  |  |  | Feedback on behaviour | Feedback process | Volition |
|  |  |  | Prompts/cues | Behavioural cueing | Volition |
|  |  |  | Credible source | Attitude towards the behaviour | Motivation |
|  |  |  | Commitment | Intentions | Volition |
| Coronado et al. (2018d) | A reminder letter and a live phone call. Significant increase in participation. | Yes | Information about health consequences | Beliefs about consequences | Motivation |
|  |  |  | Social support (unspecified) | Social influence | Motivation |
|  |  |  | Social support (practical) | Environmental context &resources | Volition |
|  |  |  | Feedback on behaviour | Feedback process | Volition |
|  |  |  | Prompts/cues | Behavioural cueing | Volition |
|  |  |  | Credible source | Attitude towards the behaviour | Motivation |
|  |  |  | Commitment | Intentions | Volition |
| Coronado et al. (2018e) | Two automated phone calls and a live phone call reminder. Significant increase in participation. | Yes | Social support (unspecified) | Social influence | Motivation |
|  |  |  | Social support (practical) | Environmental context & resources | Volition |
|  |  |  | Credible source | Attitude towards the behaviour | Motivation |
|  |  |  | Feedback on behaviour | Feedback process | Volition |
|  |  |  | Prompts/cues | Behavioural cueing | Volition |
|  |  |  | Commitment | Intentions | Volition |
| Coronado et al. (2018f) | Text message and live phone call reminder. Significant increase in participation. | Yes | Prompts/cues | Behavioural cueing | Volition |
|  |  |  | Credible source | Attitude towards the behaviour | Motivation |
|  |  |  | Social support (unspecified) | Social influence | Motivation |
|  |  |  | Social support (practical) | Environmental context & resources | Volition |
|  |  |  | Feedback on behaviour | Feedback process | Volition |
|  |  |  | Commitment | Intentions | Volition |
| Coronado et al. (2019a) | Advance notification text message. No significant difference in participation. | Yes | Advance notification | Intention | Volition |
| Coronado et al. (2019b) | Three live phone call reminders. Significant increase in participation. | Yes | Social support (unspecified) | Social influence | Motivation |
|  |  |  | Social support (practical) | Environmental context & resources | Volition |
|  |  |  | Feedback on behaviour | Feedback process | Volition |
|  |  |  | Problem solving | Beliefs about capabilities | Volition |
| Denters et al. (2013) | Faeces collection paper to aid in sampling. No significant difference in participation. | No | Adding objects to the environment | Environmental context &resources | Volition |
|  |  |  | Demonstration of the behaviour | Beliefs about capabilities | Volition |
| Deutekom et al. (2010) | Use of FIT with fewer samples needed to be taken. Significant increase in participation. | No | Remove aversive stimulus | Environmental context & resources | Volition |
| Digby et al. (2013) | Use of FIT with fewer samples needed to be taken. Significant increase in participation | No | Remove aversive stimulus | Environmental context & resources | Volition |
| Durkin et al. (2019a) | High intensity media campaign (Queensland trial). Significant increase in participation. | Yes | Information about health consequences | Perceived susceptibility | Motivation |
|  |  |  | Vicarious consequences | Beliefs about consequences | Motivation |
|  |  |  | Prompts/cues | Behavioural cueing | Volition |
|  |  |  | Information about health consequences | Beliefs about consequences | Motivation |
| Durkin et al. (2019b) | Low intensity media campaign (Western Australia trail). No significant difference in participation. |  | Information about health consequences | Perceived susceptibility | Motivation |
|  |  |  | Vicarious consequences | Beliefs about consequences | Motivation |
|  |  |  | Prompts/cues | Behavioural cueing | Volition |
|  |  |  | Information about health consequences | Beliefs about consequences | Motivation |
| Gupta et al. (2016a) | $5 incentive conditional on kit return. No significant difference in participation. | Yes | Material incentive (behaviour) | Reinforcement | Motivation |
| Gupta et al. (2016b) | $10 incentive conditional on kit return. No significant difference in participation. | Yes | Material incentive (behaviour) | Reinforcement | Motivation |
| Hewitson et al. (2011a) | GP endorsement letter by personal GP. Significant increase in participation. | Yes | Information about health consequences | Beliefs about consequences | Motivation |
|  |  |  | Credible source | Attitude towards the behaviour | Motivation |
|  |  |  | Information about health consequences | Perceived susceptibility | Motivation |
| Hewitson et al. (2011b) | Leaflet giving more explicit information on how to carry out and return the FOBT along with more CRC risk information. Significant increase in participation. | Yes | Information about health consequences | Beliefs about consequences | Motivation |
|  |  |  | Instruction on how to perform the behaviour | Beliefs about capabilities | Volition |
|  |  |  | Information about health consequences | Perceived susceptibility | Motivation |
| Hewitson et al. (2011c) | Both interventions strategies of the other trials combined. Significant increase in participation. | Yes | Information about health consequences | Beliefs about consequences | Motivation |
|  |  |  | Instruction on how to perform the behaviour | Beliefs about capabilities | Volition |
|  |  |  | Information about health consequences | Perceived susceptibility | Motivation |
|  |  |  | Credible source | Attitude towards the behaviour | Motivation |
| Hirst et al. (2017) | A text-message reminder from GP. No significant difference in participation. | Yes | Credible source | Attitude towards the behaviour | Motivation |
|  |  |  | Prompts/cues | Behavioural cueing | Volition |
| Hughes et al. (2005) | Use of FIT that does not require dietary restriction and uses fewer samples. Significant increase in participation. | No | Remove aversive stimulus | Environmental context & resources | Volition |
|  |  |  | Remove punishment | Environmental context & resources | Volition |
| King et al. (1992a) | GP endorsement letter. Significant increase in participation. | No | Information about health consequences | Beliefs about consequences | Motivation |
|  |  |  | Credible source | Attitude towards the behaviour | Motivation |
| King et al. (1992b) | GP endorsement letter with no dietary restriction given with the kit. Significant increase in participation. | No | Information about health consequences | Beliefs about consequences | Motivation |
|  |  |  | Credible source | Attitude towards the behaviour | Motivation |
|  |  |  | Remove punishment | Environmental context & resources | Volition |
| King et al. (1992c) | GP endorsement letter, no dietary restriction given with the kit, and CRC information brochure. Significant increase in participation. | No | Information about health consequences | Beliefs about consequences | Motivation |
|  |  |  | Credible source | Attitude towards the behaviour | Motivation |
|  |  |  | Remove punishment | Environmental context & resources | Volition |
|  |  |  | Information about health consequences | Perceived susceptibility | Motivation |
| King et al. (1994) | Educational brochure. No significant difference in participation. | No | Information about health consequences | Perceived susceptibility | Motivation |
|  |  |  | Information about health consequences | Beliefs about consequences | Motivation |
| Libby et al. (2011a) | Advance notification letter. Significant increase in participation. | Yes | Advance notification | Intentions | Volition |
| Libby et al. (2011b) | Sending the CRC information pack with advance notification. Instead of with FOBT. Significant increase in participation. | Yes | Advance notification | Intentions | Volition |
| Lo et al. (2014) | Sending implementation intention questions with prefilled with responses. No significant increase in participation. | Yes | Instruction on how to perform the behaviour | Beliefs about capabilities | Volition |
|  |  |  | Self-talk | Motivation | Volition |
|  |  |  | Action planning | Intentions | Volition |
|  |  |  | Reduce negative emotions | Behavioural regulation | Volition |
| Mehta et al. (2019a) | Unconditional $10 incentive included with the mailing. No significant difference in participation. | No | Unconditional material incentive | Social influence | Motivation |
| Mehta et al. (2019b) | $10 incentive conditional on FIT completion. No significant difference in participation. | No | Material incentive (behaviour) | Reinforcement | Motivation |
| Mehta et al. (2019c) | Conditional lottery with a 1-in-10 chance of winning $100 after FIT completion. No significant difference in participation. | No | Material incentive (behaviour) | Reinforcement | Motivation |
| Moss et al. (2016) | Use of FIT with no dietary restriction and fewer samples. Significant increase in participation. | No | Remove aversive stimuli | Environmental context & resources | Volition |
|  |  |  | Remove punishment | Environmental context & resources | Volition |
| Myers et al. (1991a) | Live telephone reminder call. Significant increase in participation. | No | Instruction on how to perform the behaviour | Beliefs about capabilities | Volition |
|  |  |  | Prompts/cues | Behavioural cueing | Volition |
|  |  |  | Problem solving | Beliefs about capabilities | Volition |
|  |  |  | Commitment | Intention | Volition |
| Myers et al. (1991b) | Live telephone reminder with CRC screening and information pack. Significant increase in participation. | No | Information about health consequences | Perceived susceptibility | Motivation |
|  |  |  | Information about health consequences | Beliefs about consequences | Motivation |
|  |  |  | Instruction on how to perform the behaviour | Beliefs about capabilities | Volition |
|  |  |  | Prompts/cues | Behavioural cueing | Volition |
|  |  |  | Problem solving | Beliefs about capabilities | Volition |
|  |  |  | Commitment | Intention | Volition |
| Myers et al. (1991c) | Live telephone instruction and reminder call with CRC screening and information pack. Significant increase in participation | No | Information about health consequences | Perceived susceptibility | Motivation |
|  |  |  | Information about health consequences | Beliefs about consequences | Motivation |
|  |  |  | Instruction on how to perform the behaviour | Beliefs about capabilities | Volition |
|  |  |  | Prompts/cues | Behavioural cueing | Volition |
|  |  |  | Problem solving | Beliefs about capabilities | Volition |
|  |  |  | Commitment | Intention | Volition |
| Neter et al. (2014) | Sending implementation intention questions with invitation. Significant increase in participation. | Yes | Action planning | Intention | Volition |
| O'Carroll et al. (2015a) | Health locus of control questions with invitation. No significant difference participation. | Yes | Framing/reframing | Attitude towards the behaviour | Motivation |
| O'Carroll et al. (2015b) | Health locus of control and anticipated regret questions with invitation. No significant difference participation. | Yes | Framing/reframing | Attitude towards the behaviour | Motivation |
|  |  |  | Anticipated regret | Beliefs about consequences | Motivation |
| Ore et al. (2001) | Added information about risks of CRC. No significant difference in participation. | No | Information about health consequences | Perceived susceptibility | Motivation |
| Robinson et al. (1994a) | Removing dietary restrictions. Significant increase in participation. | No | Remove punishment | Environmental context & resources | Volition |
| Robinson et al. (1994b) | Requesting fewer samples to be taken. No significant difference in participation. | No | Remove aversive stimulus | Environmental context & resources | Volition |
| Sandiford et al. (2017) | Offering alternative community drop off locations for samples. Significant increase in participation. | Yes | Adding objects to the environment | Environmental context & resources | Volition |
|  |  |  | Restructuring the physical environment | Environmental context & resources | Volition |
| Santare et al. (2015a) | Using OC-Sensor (FIT) kit. Significant increase in participation. | No | Remove punishment | Environmental context & resources | Volition |
|  |  |  | Remove aversive stimulus | Environmental context & resources | Volition |
| Santare et al. (2015b) | Using FOB Gold (FIT) kit. Significant increase in participation. | No | Remove punishment | Environmental context & resources | Volition |
|  |  |  | Remove aversive stimulus | Environmental context & resources | Volition |
| Santare et al. (2015c) | Sending advance notification letters. Significant increase in participation. | No | Advance notification | Intention | Volition |
|  |  |  | Information about health consequences | Perceived susceptibility | Motivation |
|  |  |  | Information about health consequences | Beliefs about consequences | Motivation |
| Schreuders et al. (2019) | Requiring one instead of two samples. Significant increase in participation. | No | Remove aversive stimulus | Environmental context & resources | Volition |
| Van Roon et al. (2011) | Sending advance notification letters. Significant increase in participation. | No | Advance notification | Intention | Volition |
|  |  |  | Information about health consequences | Perceived susceptibility | Motivation |
|  |  |  | Information about health consequences | Beliefs about consequences | Motivation |
| Verne et al. (1993a) | Using Early Detector Pads for testing. Significant increase in participation. | No | Adding objects to the environment | Environmental context & resources | Volition |
| Verne et al. (1993b) | Using *Early Detector Pads* for testing and not requesting dietary restrictions. No significant difference in participation. |  | Adding objects to the environment | Environmental context & resources | Volition |
|  |  | No | Remove punishment | Environmental context & resources | Volition |
| Verne et al. (1993c) | Using *Colscreen Self-Test* for testing. Significant increase in participation. | No | Adding objects to the environment | Environmental context & resources | Volition |
|  |  |  | Remove aversive stimulus | Environmental context & resources | Volition |
| Verne et al. (1993d) | Using *Colscreen Self-Test* for testing and not requesting dietary restrictions. No significant difference in participation. | No | Adding objects to the environment | Environmental context & resources | Volition |
|  |  |  | Remove aversive stimulus | Environmental context & resources | Volition |
|  |  |  | Remove punishment | Environmental context & resources | Volition |
| Wardle et al. (2016a) | Gist leaflet giving additional simplified information. No significant difference in participation | Yes | Instruction on how to perform the behaviour | Beliefs about capabilities | Volition |
|  |  |  | Information about health consequences | Perceived susceptibility | Motivation |
|  |  |  | Information about health consequences | Beliefs about consequences | Motivation |
| Wardle et al. (2016b) | Narratives of positive experiences of FOBT screening from peers. No significant difference in participation. | Yes | Information about health consequences | Beliefs about consequences | Motivation |
|  |  |  | Information about emotional consequences | Beliefs about consequences | Motivation |
|  |  |  | Vicarious consequences | Beliefs about consequences | Motivation |
|  |  |  | Information about others’ approval | Social Norms | Motivation |
| Wardle et al. (2016b) | GP endorsement letter with FOBT invitation. Significant increase in participation. | Yes | Credible source | Attitudes towards the behaviour | Motivation |
| Wardle et al. (2016d) | Enhanced reminder. Significant increase in Participation. | Yes | Information about health consequences | Beliefs about consequences | Motivation |
|  |  |  | Prompts/cues | Behavioural cueing | Volition |
| Watson et al. (2013) | Sending research questionnaire with FOBT kit. Significant decrease in participation. | No | Adding objects to the environment | Environmental context & resources | Motivation |
| White et al. (2015a) | CRC informational flyers from Cancer Research UK (CRUK). No significant difference in participation. | Yes | Information about health consequences | Beliefs about consequences | Motivation |
|  |  |  | Credible source | Attitude towards the behaviour | Motivation |
|  |  |  | Social comparison | Social norms | Motivation |
| White et al. (2015b) | CRC informational flyers from CRUK and sending sample collection aids. Significant increase in participation. | Yes | Adding objects to the environment | Environmental context & resources | Volition |
|  |  |  | Information about health consequences | Beliefs about consequences | Motivation |
|  |  |  | Credible source | Attitude towards the behaviour | Motivation |
|  |  |  | Social comparison | Social norms | Motivation |
|  |  |  | Instruction on how to perform the behaviour | Beliefs about capabilities | Volition |
|  |  |  | Demonstration of the behaviour | Beliefs about capabilities | Volition |
|  |  |  | Prompts/cues | Behavioural cueing | Volition |
| White et al. (2015c) | CRC informational flyers from CRUK and advertising campaign. Significant increase in participation. | Yes | Information about health consequences | Beliefs about consequences | Motivation |
|  |  |  | Credible source | Attitude towards the behaviour | Motivation |
|  |  |  | Social comparison | Social norms | Motivation |
|  |  |  | Prompts/cues | Behavioural cueing | Volition |
| White et al. (2015d) | CRC informational flyers from CRUK, sending sample collection aids, and advertising campaign. Significant increase in participation. | Yes | Adding objects to the environment | Environmental context & resources | Volition |
|  |  |  | Information about health consequences | Beliefs about consequences | Motivation |
|  |  |  | Credible source | Attitude towards the behaviour | Motivation |
|  |  |  | Social comparison | Social norms | Motivation |
|  |  |  | Instruction on how to perform the behaviour | Beliefs about capabilities | Volition |
|  |  |  | Demonstration of the behaviour | Beliefs about capabilities | Volition |
|  |  |  | Prompts/cues | Behavioural cueing | Volition |
