# Supplementary material for: Implementation Strategies for Interventions Aiming to Increase Participation in Mail-Out Bowel Cancer Screening Programs: A Realist Review
Source: Front Oncol. 2020 Sep 29;10:543732. doi: 10.3389/fonc.2020.543732 (PMC7550731; doi:10.3389/fonc.2020.543732)
Supplement: Supplementary file 3 [file Table_3.docx]

Supplementary File 2: Search Terms

**Pubmed**

((("fecal occult blood"[Title/Abstract] OR "faecal occult blood"[Title/Abstract] OR FOBT[Title/Abstract] OR "fecal immunochemical test" OR "faecal immunochemical test" [Title/Abstract])) AND (participat*[Title/Abstract] OR adher*[Title/Abstract] OR uptake[Title/Abstract] OR return [Title/Abstract] OR complian* [Title/Abstract]))

**Scopus**

TITLE-ABS-KEY ( "fecal occult blood" OR "faecal occult blood" OR fobt OR "fecal immunochemical test" OR "faecal immunochemical test" ) AND TITLE-ABS-KEY ( participat* OR adher* OR uptake OR return OR complian* ) )

**PsycInfo**

TI ( "fecal occult blood" OR "faecal occult blood" OR FOBT OR "fecal immunochemical test" OR "faecal immunochemical test" ) OR AB ( "fecal occult blood" OR "faecal occult blood" OR fobt OR "fecal immunochemical test" OR "faecal immunochemical test" ) AND TITLE-ABS-KEY ( participat* OR adher* OR uptake OR return OR complian* ) ) AND TI ( participat* OR adher* OR uptake OR return OR complian* ) AND AB ( participat* OR adher* OR uptake OR return OR complian* )

**CINAHL**

TI ( "fecal occult blood" OR "faecal occult blood" OR FOBT OR "fecal immunochemical test" OR "faecal immunochemical test" ) OR AB ( "fecal occult blood" OR "faecal occult blood" OR fobt OR "fecal immunochemical test" OR "faecal immunochemical test" ) AND TITLE-ABS-KEY ( participat* OR adher* OR uptake OR return OR complian* ) ) AND TI ( participat* OR adher* OR uptake OR return OR complian* ) AND AB ( participat* OR adher* OR uptake OR return OR complian* )

**Google Scholar**

allintitle: "fecal occult blood" OR "faecal occult blood" OR FOBT OR "fecal immunochemical test" OR "faecal immunochemical test" AND participate OR participation OR uptake OR return OR adhere OR adherence OR compliance OR compliant

**Proquest Theses and Dissertations**

ti("faecal occult blood" OR "fecal occult blood" OR fobs OR "fecal immunochemical test" OR "faecal immunochemical test") AND ti(participat* OR uptake OR return OR complian* OR adher*) OR ab("faecal occult blood" OR "fecal occult blood" OR fobs OR "fecal immunochemical test" OR "faecal immunochemical test") AND ab(participat* OR uptake OR return OR complian* OR adher*)
